# Human Group IIA Secreted Phospholipase A_2_ and the Prostate Cancer Tumor Microenvironment: Potential Mechanisms

**DOI:** 10.3390/ijms27167277

**Published:** 2026-08-14

**Authors:** Monavvar Andarva, Maria George Elias, Mila Sajinovic, Tara Laurine Roberts, Paul de Souza, Kieran F. Scott

**Affiliations:** 1 School of Medicine, Western Sydney University, Campbelltown, NSW 2560, Australia; 22059348@student.westernsydney.edu.au (M.A.); m.elias3@westernsydney.edu.au (M.G.E.); m.sajinovic@westernsydney.edu.au (M.S.); tara.roberts@westernsydney.edu.au (T.L.R.); p.desouza@westernsydney.edu.au (P.d.S.); 2 Ingham Institute of Applied Medical Research, Liverpool, NSW 2170, Australia; 3 Nepean Hospital Clinical School, University of Sydney, Kingswood, NSW 2747, Australia

**Keywords:** prostate cancer, tumor microenvironment, secreted phospholipase A_2_ group IIA, PLA2G2A, immune modulation

## Abstract

Human secreted phospholipase A_2_ group IIA (hGIIA) is a Ca^2+^-dependent extracellular enzyme that supports host defense and amplifies inflammation through lipid mediator generation and receptor-linked signaling. In prostate cancer (PCa), hGIIA is commonly elevated compared with benign tissue and can persist after androgen deprivation, implicating it in tumor progression. PCa is typically an immunologically “cold” disease sustained by an immunosuppressive tumor microenvironment enriched in regulatory T cells ( T*_reg_*), myeloid-derived suppressor cells (MDSCs), tumor-associated macrophages (TAMs), and pro-tumor stromal cells, alongside hypoxia and metabolic rewiring. Because hGIIA is released by innate immune cells and is inducible in macrophages, it is well positioned to strengthen inflammatory lipid networks and promote immune dysfunction within the prostate tumor microenvironment. Here, we review the PCa microenvironment and evaluate evidence linking hGIIA to tumor–stroma crosstalk, angiogenic programming, and therapy resistance, highlighting major knowledge gaps in prostate-focused immune studies.

## 1. Introduction

The phospholipase A_2_ (PLA_2_) superfamily encompasses six structurally distinct enzyme groups that cleave the *sn*-2 ester bond in phospholipids, producing free fatty acids and lysophospholipids vital for physiological functions. The first group identified in humans was secreted enzymes (sPLA_2_s) that exhibit tissue-specific expression. For example, group IB sPLA_2_ is normally expressed in the pancreas, while group IIF sPLA_2_ is expressed primarily in skin cells [1,2]. The best studied human sPLA_2_ (hGIIA, group IIA sPLA_2_, sPLA_2_-IIA, PLA2G2A) is involved in host defense against infection in healthy subjects, being expressed in and released from secretory glands such as salivary glands, Paneth cells, the prostate gland, lacrimal glands and the lactating breast. The enzyme is also stored in vesicles of innate immune cells such as mast cells, platelets and neutrophils. Its expression is inducible in other innate immune cells such as macrophages, by pro-inflammatory cytokines such as TNF and IL-1β or Toll-like receptor agonists such as LPS. Consequently, hGIIA is well positioned to be a key molecular factor in regulating innate immunity [3]. Importantly, hGIIA dysregulation is linked to immune-mediated pathologies including cancer, atherosclerosis, neuroinflammation, COVID-19, sepsis, inflammatory bowel disease, and rheumatoid arthritis [4].

Prostate cancer (PCa) is the second most common cause of cancer death in men, despite significant improvements in overall survival (OS) [5]. The current standard of care is dependent on the stage of disease and relative risk for each patient. Localized disease is treated by active surveillance, surgery or radiotherapy. Androgen deprivation therapy, androgen receptor (AR) pathway inhibitors such as abiraterone or enzalutamide and chemotherapy are commonly used to treat recurrent and metastatic disease. However, most of these patients eventually progress to castration-resistance resulting from reactivation of the androgen receptor pathway through mechanisms such as AR mutations, constitutively active AR splice variants or AR amplification. Additionally, lineage plasticity can result in selection of AR-independent tumor cell subtypes often characterized by overexpression of TP53, RB1 and PTEN loss which are treated with platinum-based chemotherapy, taxanes or etoposide. However, prognosis for these patients is poor with many surviving for under two years. Consequently, due to development of resistance to current therapies, there remains a strong need to identify novel targets for intervention [6].

hGIIA expression is significantly elevated in PCa tissues compared to normal tissues [7]. hGIIA protein expression is associated with the enzyme’s oncogenic actions, which include promoting cancer cell proliferation, motility, and invasiveness. Additionally, despite expression being androgen-dependent in normal prostate epithelium, hGIIA remains strongly expressed in tumor cells even after androgen ablation therapy, suggesting it may play a role in the persistence and aggressiveness of PCa post anti-androgen therapy. In light of these findings, hGIIA has long been identified as a potential therapeutic target in cancer, yet despite intense effort, no hGIIA inhibitors are yet approved for clinical use in any condition. Initial inhibitors potently but non-selectively inhibited the catalytic activity of hGIIA and several other sPLA_2_ enzymes. However, these inhibitors were never tested in cancers due to toxicity issues in Phase III clinical trials in patients with cardiovascular disease [3]. More recently, the cyclic peptide inhibitor of hGIIA function Kesonotide^TM^ is currently in an early-phase clinical trial (NCT06926075) for several solid tumors including PCa.

The tumor microenvironment (TME) plays a crucial role in cancer progression by facilitating tumor growth, helping cancer cells evade the immune system, and contributing to therapy resistance [8]. While there is evidence that hGIIA enhances prostate tumorigenesis through interaction with tumor cells [9], research into the relationship between the PCa microenvironment and hGIIA is limited. Theoretically, due to the aberrant expression of hGIIA in cancer, coupled with its clear role as an innate immune mediator, it is reasonable to postulate that hGIIA expression would regulate the tumor microenvironment. This review aims to summarize current knowledge of the role of the TME in PCa progression, the expression of hGIIA in the tumor microenvironment, the effect of hGIIA on immune cells and potential models of hGIIA regulation of the TME. Existing reviews have separately described the PCa TME or the broader roles of secreted PLA_2_ enzymes in cancer. The distinctive aim of this review is to integrate these fields while explicitly separating three levels of evidence: direct mechanistic evidence in PCa; supportive evidence from other tumor types or inflammatory systems; and testable but currently unproven links.

## 2. hGIIA Biology Relevant to PCa TME

### 2.1. Catalytic and Protein–Protein Interaction-Dependent Functions

hGIIA is a small, disulfide-rich, Ca^2+^-dependent extracellular enzyme. Its catalytic activity generates a bioactive free fatty acid and a lysophospholipid from susceptible phospholipid substrates. The enzyme requires mM calcium for activity, thus confining it predominantly to extracellular function. Further, the pH optimum of hGIIA ranges from 7.5 to 9.0, peaking at 8.0–8.5 [10]. The PCa TME is acidic, with pH typically ranging from 6.5 to 7.2 [11], suggesting that acidosis may reduce the catalytic function of hGIIA in the TME. In mammalian tissues, the catalytic function of hGIIA is strongly influenced by membrane composition and the viability of cells. For example, hGIIA has limited catalytic activity on phosphatidylcholine-containing phospholipids present on the surface of healthy cells, while actively hydrolyzing phosphatidylethanolamine- and phosphatidylserine-containing phospholipids on the surface of apoptotic cells. Bacterial membranes, extracellular vesicles and modified lipoproteins are generally better substrates for hGIIA than intact resting mammalian plasma membranes. Thus, it is possible that hGIIA can nevertheless indirectly provide arachidonic acid substrate to produce bioactive lipids such as eicosanoid production indirectly by remodeling extracellular lipid substrates, promoting transcellular lipid transfer, and engaging cytosolic PLA_2_-α-dependent signaling [3,4,12]. However, the production of these lipids is dependent on the presence of several arachidonic acid modifying enzymes which are differentially expressed in a cell-type-specific manner. This implies that an increase in hGIIA protein does not necessarily predict the identity or quantity of downstream lipid mediators without information on substrate availability, cellular source and local enzyme activity.

hGIIA also has catalysis-independent actions. It can bind integrins αvβ3 and α4β1, activate ERK-dependent proliferative signaling, and influence cell adhesion and migration [13,14]. In PCa, current evidence supports direct hGIIA interactions with EGFR and vimentin: EGFR binding promotes cytosolic PLA_2_-α activation and prostaglandin E_2_ (PGE_2_) production, whereas vimentin regulates intracellular trafficking and downstream stress responses [9]. These data argue against a single universal ‘hGIIA receptor’ and instead support a partner- and cell-state-dependent interaction network.

### 2.2. Cellular Sources and Inflammatory Feedback

Within a tumor, hGIIA may originate from malignant epithelial cells, infiltrating myeloid cells or other stromal compartments. Human neutrophils store active hGIIA in granules, mast cells contain hGIIA in intracellular compartments, and macrophage expression is inducible by inflammatory stimuli [15,16,17]. Secreted PLA_2_ enzymes can then alter macrophage cytokine and angiogenic profiles; in human lung macrophages, several sPLA_2_ isoforms induce TNF and VEGF-A, although the magnitude and direction of the response depend on the macrophage stimulus and enzyme isoform [18,19,20].

This biology permits feed-forward amplification. Tumor- or immune-derived cytokines can increase hGIIA expression, while hGIIA-dependent lipid and receptor signaling can enhance cytokine, chemokine and prostanoid production, which may further stimulate epithelial and stromal cells. Interactions between hGIIA and microbial or extracellular lipid substrates can additionally reshape the local lipidome and inflammatory response [21,22,23,24]. The existence of such loops is supported in inflammatory systems and several cancers, but their cellular sequence has not been demonstrated directly in human PCa tissue. Spatially resolved analysis is therefore required before assigning the dominant source or target cell in the prostate TME.

## 3. The Prostate Cancer Microenvironment Features

The prostate cancer TME is a highly dynamic network that, critically, shapes disease progression and therapeutic resistance. It comprises a complex milieu of stromal cells, immune cells, extracellular matrix, tertiary lymphoid structures (TLSs), and metastatic niches [25,26]. The PCa TME is characterized by immunosuppression, acidic and hypoxic conditions, neuronal innervation, metabolic rewiring, genomic, transcriptomic, proteomic and lipidomic alterations and resistance to systemic treatments (Table 1). These features are frequently interconnected. For example, tumor innervation can induce angiogenesis due to metabolic rewiring of endothelial cells [26].

Central to this environment is a strong immunosuppressive axis, which blunts anti-tumor immunity and facilitate tumor escape. In addition, key immunometabolites can also directly alter immune responses, for example by increasing T cell exhaustion [27]. Increasing evidence also highlights the role of neuronal innervation, where autonomic nerve signaling integrates with metabolic and immunological changes to promote tumor proliferation and invasion. Metabolic rewiring, including enhanced glycolysis and altered lipid and amino acid metabolism, not only fuels rapid tumor growth but also exacerbates immunosuppression by creating acidic and hypoxic niches [27]. Hypoxia-induced factors (HIFs) further stimulate angiogenesis and modify stromal responses, reinforcing tumor survival under stress. Genomic, transcriptomic, proteomic and metabolomic alterations in both tumor and stromal cells drive many of these processes, influencing the metabolic landscape, immune evasion, and treatment responses. Interestingly, TLSs can both support and hinder anti-tumor immunity depending on their cellular composition and cytokine profiles. Together, these interconnected features create a microenvironment that not only sustains tumor survival but also confers broad resistance to systemic treatments, highlighting the need for combinatorial therapeutic approaches targeting multiple aspects of the TME.

PCa is categorized as a “cold” tumor, viz. the tumor has a minimally responsive acquired immune system. Recent reviews have characterized the potential mechanisms by which both the tumor microenvironment and tumor cells contribute to reduced immune responses [25,28,29]. However, suppression of acquired immunity does not imply an absence of immune cells or an inactive innate compartment. Many prostate tumors contain abundant macrophages, MDSCs, neutrophils or mast cells that remain responsive to local cytokines, metabolites and tissue-damage signals. This distinction is central to the hGIIA hypothesis because hGIIA is strongly associated with innate inflammatory and lipid-remodeling circuits rather than a conventional T-cell checkpoint [25,29,30].

In PCa, direct evidence is strongest for persistent hGIIA expression in malignant cells, EGFR/vimentin-dependent signaling and tumor-cell lipid remodeling [9,31]. Evidence for regulation of immune subsets in PCa remains largely indirect and should be interpreted as a set of mechanistic hypotheses rather than established causal pathways.

**Table 1:** The key features of prostate cancer TME and their role in cancer progression.

Key Features of Prostate Cancer TME	Role in Cancer Progression	References
Immunosuppression	Mediated by regulatory T cells (T*_reg_*) and myeloid-derived suppressor cells (MDSCs) which secrete cytokines (e.g., TGF-β, IL-10, and IL-23), creating an immunosuppressive environment.	[28,32]
Acidic and Hypoxic Conditions	Result from increased glycolysis leading to lactate buildup (acidosis) and low oxygen availability (hypoxia) that upregulates hypoxia-inducible factors (HIFs), fostering angiogenesis.	[33,34,35]
Neuronal Innervation	Autonomic innervation drives tumor progression. Neurological signals enhance tumor proliferation and migration, suggesting a neuro-tumoral interaction that could be targeted therapeutically.	[36,37,38]
Metabolic Rewiring	Includes changes in glucose, lipid, and amino acid metabolism which not only support rapid tumor growth but also create a suppressive environment for immune cells.	[27,39,40]
Genomic, Transcriptomic, Proteomic and Metabolomic Alterations	Changes in both tumor and stromal cells impact the disease progression and response to therapy, with specific markers helping predict disease outcomes.	[41,42,43,44,45,46]
Tertiary Lymphoid Structures (TLSs)	Organized immune cell aggregates that can produce cytokines affecting tumor immunity; their dual role is influenced by cellular composition and the cytokines produced.	[47]
Resistance to Systemic Treatments	The pathological and inflamed microenvironment interacts with tumor cells to confer resistance to therapies, emphasizing the need for targeting the TME in treatment strategies.	[48,49,50]

## 4. Immune Cells and Their Role in the Prostate Cancer TME

The prostate cancer TME is composed of a complex array of immune and non-immune cell types (Table 2). The roles of each of the cell types have been investigated in recent years.

### 4.1. Cytotoxic T Lymphocytes (CTLs)

Although a minority of PCa cases show increased T cell infiltration, the typical intratumoral milieu is characterized by low T cell density, particularly within the CD8^+^ subset [51]. CTLs, especially CD8^+^, cells are the main anti-tumor effectors. However, many CTLs are dysfunctional/exhausted, limiting immunotherapy efficacy and enabling progression [52]. CTLs underperform in PCa since prostate tumors actively suppress CTL function. CTLs in the TME typically express high levels of exhaustion markers such as PD-1, which impair their ability to kill tumor cells [53]. The PD-1/PD-L1 pathway transmits inhibitory signals to CTLs, leading to reduced activation and proliferation [54]. Many prostate-infiltrating CD8^+^ T cells are non-functional due to upregulated PD-1, but PD-1 blockade alone is generally ineffective in most cases due to low tumor mutational burden in PCa. As a result, most prostate tumors express few neoantigens, resulting in reduced CD8^+^ T-cell activation [55,56,57]. There is little evidence that hGIIA acts directly on CTLs through a receptor. Potential hGIIA interactions are likely indirect through modulating the TME and the tumor biochemical milieu. There is limited evidence that hGIIA directly regulates CTL function in prostate cancer via a specific receptor. While it can interact with HSPGs, integrins, and PLA2R1 and impair CD8^+^ T-cell activity in other cancers [58], current data in prostate cancer suggest it acts indirectly by altering the tumor environment through EGFR/vimentin signaling, lipid changes, and eicosanoid production [9,31].

### 4.2. Regulatory T Cells (T_reg_)

T*_reg_* are abundant in the prostate-cancer microenvironment and dampen anti-tumor immunity, thereby fostering tumor growth and progression. They modulate innate and adaptive responses (e.g., by influencing NK cell function through IL-2 secretion), support cancer-cell survival directly *via* growth-factor signaling and indirectly through stromal interactions. Consequently, high intratumoral T*_reg_* infiltration is associated with poorer prognosis and increased risk of PCa recurrence [45,59,60]. Prostate tumors appear to recruit T*_reg_*. Multiple studies report enrichment of FOXP3^+^/functional T*_reg_* in prostate tissue and in peripheral blood of patients with prostate cancer. Compared with BPH (benign prostatic hyperplasia) and healthy controls, patients showed higher circulating T*_reg_* levels, and in at least one cohort T*_reg_* frequency positively correlated with PSA, collectively supporting a role for T*_reg_* upregulation in disease progression [61]. The relationship between hGIIA and T*_reg_* has not yet been well defined. However, it is plausible that hGIIA may contribute to T*_reg_* differentiation and survival through indirectly providing substrate to produce lipid mediators such as PGE_2_.

### 4.3. B Cells

A 2023 study showed that PCa patient sentinel nodes show elevated mature/activated B cells that drive B cell–directed anti-tumor immunity [62]. In metastatic castration-resistant PCa (mCRPC), androgen-deprivation as a result of castration promotes B-cell infiltration; B-cell-derived lymphotoxin (LTα_1_β_2_) activates IKK/NF-κB in tumor cells, driving progression to mCRPC [63]. Peritumoral CD20^+^ B-cell enrichment in prostate cancer is linked to adverse prognosis, serves as an independent predictor of metastasis and associates with increased recurrence risk when concentrated in tumor-adjacent glands [64,65]. As with CTLs and T*_reg_*, hGIIA has no established direct effects on B cells. Once again, effects on B cells are likely to be due to indirect modulation of the TME through bioactive lipid mediators such as prostaglandins and leukotrienes resulting in altered B-cell activation.

### 4.4. Tumor Associated Macrophages (TAM)

In PCa, evidence for the impact of TAMs on outcome is mixed. Lissbrant et al. [66] associated higher TAM volume density with shorter overall survival, whereas Shimura et al. [67] reported that high TAM counts independently predicted longer disease-free survival after surgery. In several clinical studies, TAM density is higher in PCa than in BPH and when elevated on biopsy TAMs correlate with worse clinicopathological features [68]. With tumor progression, TAMs skew from M1-like (pro-inflammatory) phenotype to M2-like (anti-inflammatory) phenotype, promoting immune evasion, proliferation, angiogenesis and invasion/metastasis [69]. The conventional M1/M2 framework is useful for describing experimental extremes but does not capture the continuous, reversible and spatially heterogeneous states of macrophages within tumors. In PCa, TAMs may co-express inflammatory, immunosuppressive and tissue-remodeling programs, with their phenotype changing in response to tumor stage and local stromal, metabolic and cytokine signals. Accordingly, the terms “M1-like” and “M2-like” are used here only when they reflect the terminology or experimental definitions of the original studies [70]. In a recent study, prostate tumor samples showed higher densities of CD68^+^ and CD163^+^ (M2) TAMs than adjacent non-cancerous tissue. CD163^+^ TAM infiltration correlated with higher Gleason score and adverse risk stratification and was positively associated with PD-L1 expression. Patients with both high CD163^+^ TAMs and high PD-L1 had shorter biochemical recurrence–free survival [71]. Though experimental studies in PCa are lacking, the mechanistic relationship between hGIIA and TAMs is likely one of the strongest hypothetical links between immune suppression and hGIIA. hGIIA-mediated induction of PGE_2_ production may aid the recruitment and polarization of immunosuppressive macrophages.

hGIIA provides a plausible macrophage-centered amplification mechanism. Macrophages can express hGIIA, and exogenous sPLA_2_ enzymes alter macrophage production of TNF, IL-6, chemokines and vascular endothelial growth factor-A (VEGF-A) through catalytic and non-catalytic pathways [15,19,20,72]. In PCa, such signaling could reinforce the CAF-macrophage-tumor feedback loops already associated with invasion and angiogenesis [73,74]. However, no study has yet shown that selective hGIIA manipulation changes human PCa TAM states in situ; this is a central knowledge gap.

### 4.5. Neutrophils

In the prostate-cancer TME, neutrophils can act as either anti-tumorigenic (N1) or pro-tumorigenic (N2), depending on their polarization state and local context [75]. The role of tumor-associated neutrophils (TANs) in the prostate TME is complex and context-dependent, with studies linking TAN enrichment to poorer prognosis in prostate cancer [76]. In contrast, a 2023 study showed that the abundance of neutrophils, basophils, mast cells, and endothelial cells showed no significant association with patient survival and supported a limited contribution of neutrophils to anti-androgen resistance [77]. In bone-metastatic prostate cancer (BM-PCa), neutrophils exert a strong anti-tumor effect. Bone-marrow neutrophils directly induce prostate-cancer cell apoptosis in vitro and in vivo, largely by suppressing STAT5 signaling. Depleting neutrophils in bone-metastasis models accelerates tumor growth (~2.3-fold), underscoring their protective role [78]. N2 neutrophil pro-tumor functions drive immune suppression, angiogenesis, and tissue remodeling, thus fueling tumor growth and metastasis [79]. They exhibit elevated arginase-1 (ARG1), dampening T-cell function, and secrete pro-tumor mediators such as VEGF, matrix metalloprotease-9 (MMP-9), and multiple chemokines [80]. Tumor-associated neutrophils can generate neutrophil extracellular traps (NETs), which provide scaffolds that promote tumor-cell migration, invasion and metastasis [81]. Recent evidence suggests that N2-polarized TANs are particularly associated with NET formation [82]. In prostate cancer, NETs are linked to faster progression and greater treatment resistance [83]. In an orthotopic PC-3/NOD-SCID model, TANs secrete more MMP-9 (matrix metalloproteinase-9) than TAMs, and TAN-derived MMP-9 promotes metastasis. Overall, neutrophils are cytotoxic to prostate cancer cells early in disease, but on progression, tumors evade this killing and the anti-tumorigenic effect of neutrophils is diminished [78,84]. Interestingly, in support of this finding, GM-CSF therapy has been shown to help control PCa in a clinical trial [85]. Evidence for a link between hGIIA and neutrophil activity in prostate cancer is limited, however. Neutrophils store hGIIA in granules that are released on stimulation. Based on what is known about neutrophil activation and the prostate TME, cytokines such as IL-8, TNF, IL-1β, colony-stimulating factors GM-CSF and G-CSF, complement factor C5a, damage-associated molecular patterns (DAMPs) are known to stimulate neutrophil activation, degranulation and reactive oxygen species (ROS) production. Given that these mediators are commonly present in tumors, it is likely that neutrophils are a major source of TME hGIIA.

### 4.6. Mast Cells

Mast cells (MCs) are abundant in the prostate cancer TME and as with other innate immune cells, exert context-dependent effects on disease, acting as tumor promoters or suppressors depending on their spatial distribution and the disease stage [86]. A study reported that MCs have stage-dependent roles in prostate cancer: they are pro-tumorigenic early, supplying MMP-9 to the microenvironment, but become dispensable at later stages as reported with neutrophils (see above) [87,88]. In prostate cancer, early reports linked high intratumoral MC density with a favorable prognosis [89]. Later work refined this view, showing that intratumoral MCs can suppress tumor growth, whereas peritumoral MCs tend to promote PCa [90]. Mast cell burden and spatial distribution can be independent prognostic indicators. Low intratumoral mast cell density predicts a higher risk of biochemical recurrence after prostatectomy, whereas high peritumoral mast cell density associates with more aggressive disease and poorer outcomes [90,91,92].

Evidence indicates that MCs can drive chemo- and radio resistance in prostate cancer by modulating the p38/p53/p21 signaling network. Accordingly, it is proposed that inhibiting these signals could mitigate treatment resistance [93]. In a mouse model, MCs also accelerated tumor growth by influencing androgen receptor (AR) activity and upregulating MMP-9, leading to the suggestion that blocking MC–AR signaling may curb tumor progression [94]. MC–tumor interactions in prostate cancer appear context-dependent. Some studies indicate MCs can promote tumor cell proliferation and EMT, processes linked to progression [95], yet their effects may vary with tumor stage and malignancy and are not fully defined. MC density and spatial distribution also matter. Higher intratumoral counts have been linked to lower cancer recurrence in some cohorts highlighting heterogeneous prognostic roles. Overall, the field needs mechanistic studies to clarify when and how MCs could be therapeutically targeted in prostate cancer [96,97]. Mast cells facilitate prostate cancer metastasis by suppressing AR in tumor cells, activating PKD (protein kinase D) to elevate C-C motif chemokine ligand 5 (CCL5)/CCL11/stem cell factor (SCF), upregulating MMP-9 to promote invasion, and downregulating SMAD4 to remodel the extracellular matrix [84].

Mast cell interactions with TME cells also influence tumor growth. MC–released tryptase amplifies CAF-driven morphological transitions in prostate epithelial cells, fostering a pro-tumorigenic phenotype [98]. A study showed that MC-derived chemokines in the prostatic epithelium and stroma recruit immune cells, leading to increased B and T lymphocytes and macrophages in the prostates of patients with BPH [99]. Also, MDSC- and MC-crosstalk enables prostate tumor immune evasion. MCs drive prostate tumor onset by activating polymorphonuclear (PMN)-MDSC–mediated immunosuppression via the CD40L–CD40 axis: CD40L on MCs engages CD40 on PMN-MDSCs to restore their suppressive function. This pathway represents a promising target to overcome tumor immune tolerance [100]. As with other immune cell-types, the relationship between hGIIA and mast cells is underexplored. Nonetheless, there is a strong biological rationale for hGIIA involvement in mast-cell function in PCa. As with neutrophils, mast cells store hGIIA in granules, which is released on activation. Mast cells are major producers of prostaglandin D_2_ (PGD_2_), which hGIIA may indirectly enhance production of through stimulation of arachidonic acid production, resulting in pro-tumorigenic or anti-tumorigenic function depending on which of two PGD_2_ receptors are stimulated or whether PGD_2_ is further metabolized to PGJ_2_. Specific data in prostate cancer is lacking [101,102].

### 4.7. Natural Killer (NK) Cells

Single-cell RNA-sequencing showed that normal prostate contains two NK subsets, mirroring blood and other tissues: CD56^dim^CD16^+^ conventional NK cells and CD56^bright^CD16^neg^ tissue-resident NK cells. CD16^+^ NKs show higher activation/cytotoxicity gene expression than CD16^−^ cells, whereas CD16^−^ tissue-resident NKs exhibit reduced cytotoxicity but heightened cytokine/chemokine programs [103]. Greater NK cell infiltration in prostate tumors is linked to better outcomes. Higher NK cell density in tumor tissue correlates with longer overall survival and a lower risk of recurrence; patients with abundant intratumoral NK cells at prostatectomy have reduced biochemical recurrence. In contrast, NK cell dysfunction is consistently associated with poorer clinical outcomes across disease stages [104,105,106]. In prostate cancer, circulating NK cells display an exhausted phenotype—decreased natural killer group 2, member D (NKG2D) with increased programmed cell death protein 1 (PD-1)/T-cell immunoglobulin and mucin domain-containing protein-3 (TIM-3)—and show markedly reduced degranulation. Functionally, they can undermine anti-tumor macrophage activity by secreting CCL2 and IL-10, which recruit monocytes and drive M2 polarization [107]. In prostate tumor models, hypoxia (a hallmark feature of solid tumors including PCa) downregulates the expression of major histocompatibility complex class I chain-related proteins A and B (MICA/MICB) and UL16-binding proteins (ULBPs), while also upregulating PD-1, thus diminishing NK cell mediated cytotoxicity. Notably, inhibition of JAK1/2 or STAT3 can reduce PD-L1 levels, and pairing these agents with PD-L1 blockade more effectively restores NK-cell function in cell culture models [108,109,110,111]. In PCa, circulating extracellular vesicles (EVs) are increased and suppress NK-cell activity (NKA), chiefly via NKG2A signaling. After prostatectomy, NKA rises, NKG2A on NK cells declines, and EV NKG2D ligands increase—identifying preoperative NKG2A and post-operative EV NKG2D ligands as key regulators and therapeutic targets [112]. In metastatic prostate cancer, patients whose peripheral NK cells show higher activating-receptor expression and greater cytotoxicity have longer time for castration resistance and OS. The activating receptors NKp46 and NKp30 best predicted outcomes, and tumor recognition depended on NKp46, NKG2D, DNAM-1 (NKp30 to a lesser extent), supporting NK-based biomarkers and NK-enhancing therapies [105]. NK cells preferentially kill prostate cancer stem–like cells through the TRAIL–DR5 pathway, highlighting a strategy to eliminate cancer-initiating cells and help prevent recurrence and progression [113].

There is currently little direct evidence linking hGIIA to NK cell function in PCa. It is plausible that elevated hGIIA may make an indirect contribution to an immunosuppressive environment that suppresses NK cell cytotoxicity through amplification of the production of eicosanoids such as PGE_2_.

### 4.8. Myeloid-Derived Suppressor Cells (MDSCs)

MDSC recruitment and expansion in PCa TME occurs through several mechanisms. For example, Hippo–YAP signaling, commonly hyperactive in solid tumors, elevates CXCL5, drawing MDSCs into the tumor via the CXCL5–CXCR2 axis [114,115]. Chromodomain helicase DNA binding protein 1 (CHD1), a key tumor suppressor, likewise shapes myeloid influx. Its loss drives IL-6–dependent MDSC recruitment and shows a positive correlation with CD15 (a PMN-MDSC marker) [116]. Complementing these pathways, PTEN loss in murine PCa amplifies inflammatory cues (increase in CSF-1, IL-1β) and results in pronounced MDSC infiltration [117]. Tumor hypoxia further sustains MDSC accumulation, whereas hypoxia-targeted therapy can induce durable reductions in these cells [118].

MDSCs shape the PCa microenvironment by promoting tumor growth and broad immune suppression. They activate multiple signaling pathways and secrete immunomodulatory factors that inhibit NK cells, macrophages, dendritic cells, and cytotoxic T cells, while inducing T*_reg_* and skewing macrophages toward an M2 phenotype. Beyond immune effects, MDSCs foster tumor initiation, migration, and metastasis through crosstalk with CAFs, endothelial cells, and osteoclasts [119]. Circulating MDSCs appear to be a useful biomarker of prostate cancer burden. Their levels help distinguish metastatic disease from localized PC and from cancer-free men [120]. They correlate with PSA and with metastatic status [121,122]. When elevated after therapy, they predict poorer overall survival in randomized studies [123,124].

MDSCs are classified by origin into two principal subsets: monocytic (Mo-MDSCs) and granulocytic/polymorphonuclear (PMN-MDSCs) [125]. Which MDSC subset is most relevant remains unsettled, largely due to variability in methods and limited standardization. Some work in mCRPC identifies prognostic value specifically for circulating Mo-MDSCs [126] and other PCa studies have emphasized Mo-MDSCs over PMN-MDSCs [123,126,127].

Tumor-infiltrating PMN-MDSCs upregulate IL-1β and IL-23a [128]; IL-1β–mediated immunosuppression is recognized in other cancers [129], and IL-23 from PMN-MDSCs—so far documented only in PCa—can preserve androgen receptor function under androgen deprivation, fostering survival and castration resistance [130]. Castration resistance may also be driven by IL-8–dependent recruitment of PMN-MDSCs [128]. MDSCs suppress anti-tumor immunity through several pathways. By expressing arginase-1 (ARG1) and inducible nitric oxide synthase (iNOS), they deplete L-arginine, an amino acid essential for T cell proliferation and function, in the TME, thereby impairing T-cell activation and diminishing IL-2 and IFN-γ production [131,132,133]. STAT3 drives MDSC development and suppressive function. In the TME, IL-6, IL-1β, IL-10, GM-CSF, and VEGF activate pSTAT3, and inhibiting pSTAT3 in an orthotopic PCa model blocks tumor growth and metastasis [134]. GM-CSF from PCa cells expands MDSCs via pSTAT3. These MDSCs secrete IL-6/IL-1β/IL-10, which induces immunosuppression [127]. TLR9 (Toll-like receptor 9) engagement in prostate cancer stimulates MDSC STAT3 activity, fostering AR-indifferent tumor growth and self-renewal [135,136,137]. Also, studies implicate PGE_2_ in inducing MDSCs and enhancing their suppressive capacity in cancer. PGE_2_ boosts GM-CSF/IL-6–mediated differentiation of M-MDSCs [138,139]. PGE_2_-conditioned M-MDSCs are associated with inhibited T cell proliferation, expansion of alloreactive Th2 cells, and diminished Th17 and cytotoxic T-cell development. In prostate cancer, PGE_2_ is elevated and linked to COX-2 inhibition [140,141], increased M-MDSC induction, and consequent suppression of cytotoxic T cells [119,142]. Additionally, MDSCs also generate reactive nitrogen species (RNS) that nitrate tyrosine residues on key T-cell proteins. In particular, nitration of lymphocyte-specific protein tyrosine kinase (LCK), an initiating kinase in T-cell receptor signaling, at Tyr394 impairs T-cell activation and limits proliferation [143]. Although hGIIA has not yet been directly shown to regulate MDSC function in the prostate cancer TME, existing evidence supports a plausible indirect link: hGIIA can activate EGFR/cPLA_2_α signaling and PGE_2_ production in prostate cancer cells, while PGE_2_ is a known inducer of MDSC differentiation and immunosuppressive function [144].

### 4.9. Dendritic Cells (DCs)

In the prostate cancer TME, dendritic cells (DCs) are metabolically constrained and functionally impaired. Like T cells, DCs depend on glycolysis for activation and antigen presentation, but nutrient deprivation limits glucose uptake, driving dysfunction. Elevated lactate and other byproducts further block DC maturation, blunting T-cell priming [145,146]. Tumors attract immature DCs via inflammatory chemokines, but once on site, a suite of immunosuppressive cues blocks their maturation and function. Single-cell RNA-seq in prostate cancer shows tumor-infiltrating DCs downregulate key surface molecules (e.g., GPR34, SLCO2B1), consistent with weakened antigen presentation [147]. Prostate cancer tissue contains far fewer DCs than benign tissue. Across studies, total, mature, and functional DC populations are markedly reduced in PCa. This deficit increases with tumor aggressiveness. Notably, conventional type 1 dendritic cells (cDC1s), the subset essential for cross-presenting tumor antigens to CD8^+^ T cells, are especially depleted in patients relative to healthy donors [148,149,150]. Monocyte-derived DCs (Mo-DCs) from prostate cancer patients display markedly reduced maturation marker expression (HLA-DR, CD80, and CD86) relative to healthy donors, resulting in weakened T-cell priming and impaired anti-tumor immunity [151]. Prostate cancer-derived exosomes potently suppress DC function. Loaded with immunosuppressive cargo such as PGE_2_, they induce CD73 on DCs, driving adenosine-mediated inhibition. Exosomes from castration-resistant PCa further reduce natural killer group 2 member D (NKG2D) on NK and CD8^+^ T cells when presented via DCs, dampening cytotoxic responses [152,153]. Interleukin-6 (IL-6) is abundantly produced in prostate cancer by tumor, stromal, and immune cells. Tumor and stromal IL-6 activate STAT3, elevate PD-L1 on dendritic cells, and blunt anti-tumor immunity. IL-6 also downregulates major histocompatibility complex (MHC) class II on bone marrow-derived DCs and suppresses their pro-inflammatory functions [154]. PGE_2_ counteracts prostate TME–induced DC migration defects. Murine prostate cancer cell line RM-1-conditioned medium impaired DC chemotaxis to CCL19 and lymph-node homing by activating LXRα and suppressing CCR7; PGE_2_ restored migration by upregulating CCR7, inhibiting LXRα, and boosting matrix metalloproteinase 9 (MMP9) and Notch1 signaling. In RM-1 tumor–bearing mice, PGE_2_ increased intratumoral DCs and T cells and reduced tumor growth [155].

While there is significant evidence that PGE_2_ suppresses DC function, an indirect link between hGIIA and reduced DC function is yet to be determined. Such a link is plausible given the known association of elevated hGIIA and amplification of PGE_2_ production. In addition, it is possible that hGIIA may result in enhanced lipid accumulation in DCs by a vimentin-dependent mechanism as recently established in PCa cells [31].

### 4.10. Cancer-Associated Fibroblasts (CAFs)

CAFs, the dominant stromal population in the prostate TME, drive disease by coordinating extensive crosstalk with immune and non-immune cells. Through these interactions, CAFs promote tumor growth, reshape immunity, foster therapy resistance, and enhance metastatic potential, making them central architects of prostate cancer progression [156]. Two major CAF subtypes have been identified in PCa: αSMA^+^ CAV1^+^ CAFs (C0) and FN1^+^ FAP^+^ CAFs (C1), with CAFs-C1 associated with castration resistance and poor prognosis [157]. CAF–tumor crosstalk in prostate cancer is IL-6–dependent. Carcinoma-derived IL-6 activates fibroblasts, which then secrete MMPs that drive epithelial–mesenchymal transition (EMT) in cancer cells. This EMT amplifies tumor growth, enables spontaneous metastasis, and endows carcinoma cells with stem-like traits including upregulated stem-cell markers, prostasphere formation, and self-renewal, thus linking CAF activity to increased aggressiveness and metastatic potential [158]. In a 2025 study, β-catenin and C-X-C motif chemokine receptor 4 (CXCR4) were strongly co-localized in xenograft tumors, indicating that CAFs enhance prostate-cancer-stem-cell (PCSC) stemness and drive castration-resistant prostate cancer (CRPC) progression by upregulating Wnt3a and stromal cell-derived factor-1 (SDF-1), thereby activating Wnt/β-catenin and SDF-1/CXCR4 signaling. These pathways present actionable targets for CRPC therapy [159]. Reciprocal signaling between CAFs and prostate cancer cells drive progression. Tumor cells activate fibroblasts by releasing IL-6, TGF-β, PDGF, and FGF2. In response, CAFs secrete growth factors, cytokines, and chemokines that boost tumor proliferation, survival, migration, and invasion. Notable paracrine mediators include TGF-β (activating EMT and stemness), FGFs (activating RAS/MAPK, PI3K/AKT), HGF (activating c-MET), CXCL12 (activating CXCR4), and IL-6 (activating JAK/STAT3).

Beyond soluble cues, direct CAF–tumor cell contact further amplifies these pro-tumorigenic effects [160,161]. Also, it has been shown that CAFs with high YAP1 expression drives prostate cancer progression as follows. CAFs with high YAP1 promote epithelial tumor cell proliferation and are linked to distant metastasis. Silencing YAP1 or SRC in CAFs blunts the pro-proliferative/invasive effects of CAF-conditioned media on prostate cancer cells, indicating these tumor-promoting actions rely on CAF paracrine signaling via YAP1/SRC [162,163]. Additionally, a multi-omics study in advanced PCa has shown an intercellular communication between FAP^+^ fibroblasts and SPP1^+^ macrophages in PCa TME [74]. This study identifies promyelocytic leukemia zinc finger protein (PLZF) as a tumor suppressor that blocks STAT3 phosphorylation by transcriptionally upregulating the tyrosine phosphatase SHP-1, thereby inhibiting JAK–STAT3 signaling. PLZF is reduced in high-grade prostate cancer versus benign tissue, and its loss enhances tumor cell proliferation, survival, migration, and invasion. Fibroblast-derived chemokine ligand 3 (CCL3) suppresses PLZF, further driving invasiveness. Therefore, CAF–tumor interactions amplify PCa malignancy by silencing PLZF [164].

Fibroblast hGIIA expression is well documented in other cancers such as pancreatic, colorectal, breast and gastric cancers, particularly in response to cytokine stimulation suggesting that PCa fibroblasts are likely to be capable of hGIIA expression. Single-cell and spatial transcriptomic data from oral squamous cell carcinoma identify a *PLA2G2A*-expressing subpopulation of CAFS that have both pro-tumorigenic or anti-tumorigenic effects on an aggressive epithelial cell population depending on the relative activation of IGF-1 signaling (tumor promoting) or IGF-1 receptor signaling (tumor suppressing) in the epithelial cells [165]. While single-cell approaches have not yet identified hGIIA as a marker of a CAF population, these studies raise an interesting possibility that may explain the pro- and anti-tumorigenic roles of hGIIA and warrant further investigation.

### 4.11. Tumor Endothelial Cells

Tumor endothelial cells (TECs) drive pathological angiogenesis in PCa, creating disorganized, dilated, and poorly functioning vessels under strong VEGF signaling, especially in advanced/metastatic disease. Single-cell RNA-seq reveals diverse TEC subtypes (arterial, venous, immature, and angiogenic tip cells), each playing distinct roles in building and remodeling the tumor vasculature [166,167,168]. In a CRPC model, endothelial cells drive prostate cancer invasion and metastasis by secreting C-C motif chemokine ligand 5 (CCL5), which suppresses AR expression and induces autophagy in tumor cells [169]. Autophagy accelerates focal adhesion disassembly, enhancing invasiveness. Blocking CCL5/CCR5 signaling and autophagy markedly reduces metastasis in vivo, highlighting endothelial cell–mediated CCL5–AR–autophagy as a therapeutic axis for mCRPC. ECs in prostate cancer exhibit striking functional plasticity and pro-tumor activity. Within the bone metastatic niche, they can transdifferentiate into osteoblast-like cells, potentially fostering osteogenic lesions and tumor colonization [170]. Also, EC-expressed galectin-1 (Gal-1) suppresses T cell trans-endothelial migration driven by PCa cells, aiding immune evasion [171]. In ECs, the endosomal phagocyte NADPH oxidase 2 (NOX2), critical for antigen presentation and immune regulation, generates reactive oxygen species that regulate EC proliferation and angiogenic signaling, sustaining the aberrant neo-vasculature that supports tumor growth and dissemination [172].

hGIIA expression as measured by published single-cell analysis in PCa appears confined to a small subset of activated endothelial cells. ECs may also be responsive to hGIIA secreted from other stromal cells though poorly characterized, potential mechanisms of hGIIA function include the promotion of angiogenesis through the production of eicosanoids, leukotrienes, thromboxanes and lysophosphatidic acid resulting in promotion of endothelial proliferation. It is therefore possible that stromal hGIIA may have more impact on endothelial cell function than EC expression.

**Table 2:** Immune cells of the prostate cancer TME, highlighting their roles, mechanisms of action and interactions.

Cell Type	Mechanisms and Interactions	References
**Acquired immune cells**		
Cytotoxic T Lymphocytes (CTLs)	CTLs recognize and kill tumor cells presenting specific antigens. However, in the TME, their activity can be suppressed by regulatory cells and inhibitory cytokines released by TAMs and other suppressor cells. CTLs are not commonly activated in prostate tumors relative to other cancers. Negative correlation between CTL infiltration and tumor outcomes.	[32,173,174]
Regulatory T Cells (T*_reg_*)	T*_reg_* cells suppress the immune response against the tumor by inhibiting CTLs and NK cells. They use mechanisms such as the secretion of immunosuppressive cytokines (e.g., TGF-β and IL-10) and direct cell–cell interactions that dampen inflammatory responses and promote tumor immune tolerance in both primary and metastatic sites.	[175,176,177,178]
B Cells	B cells in the prostate tumor microenvironment (TME) influence both tumor progression and anti-tumor immunity through antibody production, antigen presentation, and cytokine secretion. Certain B-cell subsets can either promote tumor growth or enhance anti-tumor responses. Increased CD20 expression and B-cell presence in tumor-adjacent areas often predict disease aggressiveness and prognosis. Interactions between B cells and other immune cells, like T cells and macrophages, are vital for modulating the immune response and treatment outcomes.	[59,63,179]
**Innate immune cells**		
Tumor-associated macrophages (TAMs)	TAMs secrete cytokines, chemokines and growth factors that aid tumor cell proliferation, angiogenesis, and immunosuppression. They also interact with other immune cells to suppress anti-tumor responses, often through the production of IL-10 and TGF-β, which inhibit cytotoxic T cell function. M1 and M2 macrophages are associated with and predictive of adverse biochemical recurrence and poor prognosis.	[28,59,73,84,178,180,181]
Neutrophils	Pro-tumoral neutrophils (N2) promote tumor growth, angiogenesis, and metastasis by releasing cytokines, growth factors, reactive oxygen species (ROS), and enzymes that degrade the extracellular matrix. Anti-tumoral neutrophils (N1) enhance immune responses by recruiting other immune cells and directly attacking tumor cells. The tumor microenvironment influences neutrophil polarization towards either pro-tumoral or anti-tumoral roles. High neutrophil numbers and high neutrophil/ lymphocyte ratio correlate with and are biomarkers for poor prognosis.	[76,78,79,81,82,182]
Mast Cells	Mast cells in the prostate tumor microenvironment promote tumor growth by releasing substances that enhance blood vessel formation, modify the extracellular matrix, and suppress immune responses and interacting with cancer cells and other stromal cells. However, it remains controversial whether mast cells are pro- or anti-tumorigenic in prostate cancer. Mast cell infiltration can result in resistance to radiotherapy or chemotherapy sensitivity.	[87,94,95,96,183,184]
Myeloid-Derived Suppressor Cells (MDSCs)	MDSCs suppress T cell activation and proliferation through various mechanisms, including the production of arginase, nitric oxide, and reactive oxygen species. They also promote the expansion of T_reg_ within the TME and modulate macrophage polarization towards a tumor-promoting phenotype.	[100,114,185,186]
Dendritic Cells (DCs)	In the TME, tumor-derived factors can alter DC maturation and function, resulting in impaired T cell activation and a more tolerogenic, rather than immunogenic environment.	[178,187]
**Non-immune cells**		
Cancer-associated Fibroblasts (CAFs)	Cancer-associated fibroblasts (CAFs) are the dominant stromal population in the prostate cancer tumor microenvironment (TME) and orchestrate almost every hallmark of disease progression, from tumor initiation to therapeutic escape.	[73,156,188]
Tumor Endothelial Cells	Endothelial cells shape prostate cancer tumor microenvironment alongside fibroblasts and macrophages. By releasing key factors, they drive angiogenesis, metastasis, and drug resistance, making them prime targets for therapeutic intervention. Prostate cancer mainly spreads to bone via an endothelial-to-osteoblast transition.	[167,170]

## 5. Human Group IIA Secreted Phospholipase A_2_ (hGIIA) in the Tumor Microenvironment (TME)

Secreted phospholipases A_2_ play a significant role in shaping the TME by influencing both immune and inflammatory responses. These enzymes have two main ways of acting, first, through their ability to break down phospholipids, a function requiring millimolar calcium and thus largely confined to extracellular spaces and second, through their non-catalytic function in binding cell surface receptors, with subsequent internalization and intracellular function [12]. Among sPLA_2_ groups, hGIIA has a major role in cancer biology, particularly in how tumors interact with the TME. This role may either help and hinder cancer growth depending on the type of cancer [189].

In normal prostate epithelium and androgen-sensitive LNCaP cells, hGIIA expression is androgen responsive, whereas its expression persists in malignant cells after androgen ablation and is elevated in androgen-independent LNCaP-AI cells [7,21]. In advanced PCa, hGIIA expression may therefore become sustained through AR-independent HER/ERBB–PI3K–Akt–NF-κB signaling [21,22,23]. More recent studies further show that hGIIA regulates EGFR–cPLA_2_α–PGE_2_ signaling and vimentin-dependent intracellular trafficking in castration-resistant and androgen-independent models [9], as well as vimentin-dependent lipid-droplet remodeling in androgen-independent prostate cancer cells [31]. These findings support hGIIA activity across different androgen-response states, but whether hGIIA directly promotes the acquisition of castration resistance remains unresolved.

### 5.1. Pro-Tumorigenic Role of hGIIA

• Inflammation and Tumor Progression: hGIIA is typically upregulated in a variety of cancers, including prostate, colorectal, and breast cancers [190,191,192,193,194]. Its enzymatic activity breaks down lipoproteins and generates eicosanoids from arachidonic acid, which are key mediators of inflammation. Chronic inflammation in the tumor microenvironment can drive cancer progression by encouraging cell proliferation, angiogenesis, and metastasis [195]. In colorectal cancer, hGIIA has been linked to pro-inflammatory signaling pathways that promote tumor growth through the upregulation of cyclooxygenase-2 (COX-2) and prostaglandin production [196,197,198,199].
• Proliferation and Metastasis: hGIIA is associated with the activation of mitogenic signaling pathways, such as the extracellular signal-regulated kinase (ERK) pathway, which enhances cell proliferation and migration [13,200]. Overexpression of hGIIA in some cancers correlates with more aggressive tumor phenotypes, increased invasiveness, and metastasis [190]. hGIIA promotes metastasis in breast cancer by inducing EMT, a key step in the metastatic cascade [201].
• Angiogenesis: hGIIA contributes to the angiogenic process by promoting the production of lipid mediators that facilitate endothelial cell migration and blood vessel formation, creating a favorable environment for tumor growth [202]. hGIIA-derived lipid mediators, such as phosphatidic acid (PA), have been implicated in angiogenesis in prostate cancer [21,203,204].

### 5.2. Anti-Tumorigenic Role

Tumor Suppression in Certain Cancers: Interestingly, hGIIA can have tumor-suppressive functions depending on the tumor type and microenvironment. In some contexts, it appears to limit tumor growth by directly targeting cancer cells and triggering apoptosis [205,206]. For example, a recent PCa study showed that levels of hGIIA mRNA, as measured by quantitative RT-PCR, were associated with lower prostate-specific antigen (PSA) levels in 108 patient prostate tissues following radical prostatectomy [207]. Table 3 shows reported anti-/pro-tumorigenic role of hGIIA in many different cancer types.

## 6. The Effect of hGIIA on Prostate TME Immune Modulation

Some immune cells can secrete hGIIA, e.g., macrophages [15,240,241], mast cells [16,241,242] and neutrophils [17]. hGIIA can modulate the immune response by affecting immune cells within the tumor microenvironment, for example, by promoting the recruitment of immunosuppressive cells like MDSCs and T*_reg_*, which inhibit anti-tumor immunity [24]. sPLA_2_s activate macrophages via enzymatic and non-enzymatic mechanisms, promoting pro-inflammatory lipid mediators, cytokines, chemokines, and angiogenic factors. Notably, hGIIA and other sPLA_2_ isoforms (except hGXIIA) induced significant TNF-α and VEGF-A production in human lung macrophages, supporting their role in both M1-like and M2-like macrophage activation [18,19]. sPLA_2_ activity generates lipid mediators like lyso-PC and PGE_2_, enhancing dendritic cell (DC) maturation. While DCs lack endogenous sPLA_2_, transcellular activation by sPLA_2_ from neighboring cells, such as macrophages, amplifies immune responses. Notably, sPLA_2_-IIA is highly expressed in inflammatory lesions, highlighting its role in immune activation [243,244]. sPLA_2_-IIA promotes the differentiation of mononuclear cells, enhancing their adhesion and migration abilities. This suggests a novel role for sPLA_2_-IIA as a bridge between innate and adaptive immunity [14].

In some cancers, hGIIA helps tumors evade immune surveillance by reducing the efficacy of natural killer (NK) cells and cytotoxic T lymphocytes (CTLs) like in Pancreatic Ductal Adenocarcinoma (PDAC)—specific metabolic CAF subset (meCAFs) expressing PLA2G2A increases total CD8^+^ T cell accumulation yet impedes their intratumoral infiltration. By activating MAPK/ERK and NF-κB pathways, these PLA2G2A^+^ meCAFs weaken CD8^+^ T cell anti-tumor function [58].

As previously mentioned, sPLA_2_-IIA contributes to the generation of free fatty acids and lysophospholipids, which are important for various cellular signaling pathways. These lipid mediators can further influence the activation and function of both DCs and T cells, creating a feedback loop that enhances the immune response [243,245]. hGIIA acts as an “inflammation amplifier” within the tumor microenvironment by binding to receptors on immune cells (e.g., macrophages, mast cells) and driving the release of cytokines (TNF, IL-1β, IL-6) [20]. These cytokines activate epithelial cells to increase expression of both hGIIA and cPLA_2_-α, thereby promoting the production of pro-tumorigenic metabolites such as eicosanoids and lysophosphatidic acid (LPA) [21,246], resulting in a positive feedback loop.

Collectively, the available evidence supports a model in which hGIIA acts as an extracellular amplifier of lipid and inflammatory signaling within the PCa TME. hGIIA released by tumor cells, macrophages, neutrophils or mast cells could increase lipid-mediator production, stimulate myeloid cytokine release, activate stromal and endothelial cells and indirectly reduce effective anti-tumor immunity. These processes could promote macrophage-driven inflammation, MDSC activity, CAF activation, angiogenesis and T-cell dysfunction.

Nevertheless, most immune effects attributed to hGIIA in PCa remain proposed rather than demonstrated. Future studies should identify the cellular sources of active hGIIA in human PCa, distinguish catalytic from non-catalytic effects, and determine whether selective hGIIA inhibition alters macrophage states, MDSC abundance, dendritic-cell function, T*_reg_* recruitment, NK-cell activity, CD8^+^ T-cell infiltration or stromal and vascular organization. Spatial transcriptomics, spatial proteomics, activity-based probes and cell-specific genetic models will be particularly important for establishing whether hGIIA is a causal regulator of the PCa immune microenvironment rather than simply a marker of inflammation.

## 7. Conclusions

**Figure 1:**
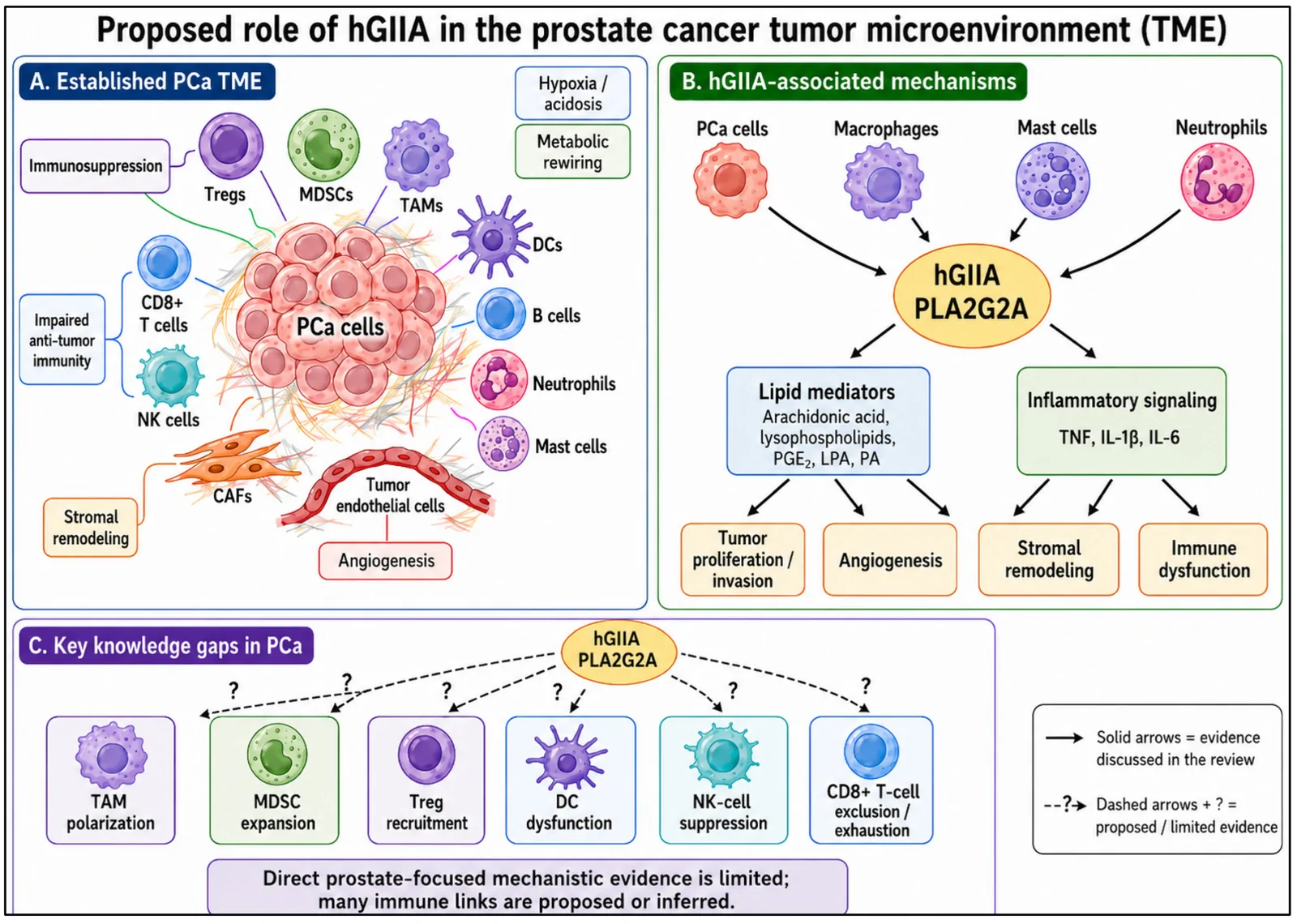
**Potential hGIIA involvement in the prostate cancer TME**. Established PCa TME components (**A**) are shown alongside hGIIA-associated lipid and inflammatory signaling pathways (**B**). Dashed arrows indicate proposed immune-cell interactions where direct prostate-focused evidence remains limited (**C**).

The prostate cancer microenvironment is increasingly recognized as a major determinant of tumor progression, immune escape, and therapeutic resistance. Rather than functioning as a passive background, the prostate TME integrates immune suppression, stromal remodeling, hypoxia, altered lipid metabolism, angiogenesis, and tumor–immune crosstalk into a dynamic network that supports disease persistence [7,10,25]. Within this context, hGIIA represents a biologically plausible mediator linking inflammation, lipid signaling, and immune dysfunction in prostate cancer (Figure 1A).

Current evidence indicates that hGIIA is elevated in prostate cancer tissues compared with benign prostate tissue and may contribute to tumor progression through effects on cancer-cell proliferation, survival, invasion, lipid mediator production, and angiogenic signaling [5,78,188,192]. Because hGIIA is secreted by innate immune cells and can be induced by inflammatory stimuli, it is also well positioned to influence the immune architecture of the prostate TME. Through enzymatic generation of free fatty acids and lysophospholipids, as well as receptor-linked signaling, hGIIA may amplify inflammatory lipid networks that favor tumor-associated macrophage activation, stromal remodeling, angiogenesis, and immune suppression [168,228,233] (Figure 1B). While hGIIA is central to many cellular functions, there is little evidence, at present, to suggest causal links to specific immune-related cell populations.

However, the role of hGIIA in cancer is clearly context-dependent. Although many studies support pro-tumorigenic functions in prostate and other cancers, emerging evidence also suggests that hGIIA expression may associate with favorable prognosis in selected prostate cancer cohorts [193]. This duality indicates that hGIIA should not be considered a universally oncogenic factor. Instead, its biological effect is likely shaped by tumor stage, androgen signaling status, cellular source, enzymatic activity, lipid substrate availability, stromal composition, and immune contexture.

A major limitation in the current field is the lack of direct mechanistic studies examining hGIIA within the prostate cancer immune microenvironment (Figure 1C). Most proposed links between hGIIA and immune suppression in prostate cancer remain inferential, based on evidence from other cancers, inflammatory disease models, macrophage biology, or broader lipid mediator signaling. Future studies should therefore define which prostate tumor and stromal cell populations express hGIIA, how its expression changes during androgen deprivation and castration resistance, and whether hGIIA directly regulates macrophage polarization, MDSC expansion, T*_reg_* recruitment, dendritic-cell dysfunction, NK cell suppression, or CD8^+^ T-cell exclusion.

Overall, hGIIA provides a potential molecular bridge between inflammation, lipid metabolism, stromal activation, and immune suppression in prostate cancer. Clarifying whether hGIIA has a role in the prostate TME could determine the influence of hGIIA on tumor progression.

## Figures and Tables

**Table 3:** Role of hGIIA in different cancer types.

Cancer Type	Pro-Tumorigenic Mechanism and Consequences	References
Colorectal Cancer	*Pro-tumorigenic* Tumor hGIIA expression associated with apoptotic cells. Higher expression in peritumoral cells. Increased xenograft tumor size on expression of murine (m)PLA2G2A.	[192,198,208,209]
	*Anti-tumorigenic* mPLA2G2A suppresses tumorigenesis in mouse models possibly through its bactericidal properties of modulating the inflammatory response. Genetic ablation of mPLA2G2A improves recovery from inflammation but increases cancer susceptibility through release from homeostatic Wnt inhibition.	[190,210]
Prostate Cancer	*Pro-tumorigenic* hGIIA expression increases with PCa grade. Tumor progression promoted by creating a positive feedback loop in the EGFR/HER2 network maintaining a cancer stem cell phenotype. Selective inhibition of hGIIA signaling through EGFR and vimentin binding inhibits xenograft tumor growth through increased PGE_2_ production and apoptosis.	[7,22,23,190,211,212,213]
	*Anti-tumorigenic* hGIIA overexpression associated with good prognosis in a radical prostatectomy cohort of 108 patients followed for 163 months. Bioinformatic analysis of expression databases indicates low expression of hGIIA associated with poorer outcomes.	[207,214,215]
Breast Cancer	*Pro-tumorigenic* hGIIA expression elevated in advanced breast cancer tissue and nipple aspirates. Low levels correlate with decreased malignancy potency, less aggressive tumor characteristics and longer patient survival. hGIIA knockdown in culture reduces numbers, tumorigenic potential, metastatic potential, increased differentiation potential of breast cancer stem cells and reduces EGFR/JNK/ c-JUN-c-Fos signaling.	[190,193,201,216]
	*Anti-tumorigenic* hGIIA epigenetically silenced via histone deacetylation in breast cancer cell lines. Oncomine data indicated both up and downregulation of hGIIA expression in tumor biopsies.	[217]
Gastric Cancer	*Pro-tumorigenic* No Significant Reports	No Significant Reports
	*Anti-tumorigenic* hGIIA is associated with prolonged survival and less frequent metastases. Co-expressed with EphB and is also a target for wnt signaling inhibiting invasion and metastasis.	[218,219,220,221,222]
Pancreatic Cancer	*Pro-tumorigenic* hGIIA regulates CD8^+^ T cells and is a marker of a CAF subset that facilitates immune escape in PDAC. Oncogenic K-Ras upregulates hGIIA in pancreatic cancer cells. Elevated hGIIA in pancreatic cancer tissue correlates with poor survival. Elevated hGIIA promotes cancer cell survival by reducing lipid peroxidation, enhancing energy metabolism through *de novo* fatty acid synthesis.	[58,223,224,225]
	*Anti-tumorigenic* Elevated hGIIA correlates with increased fibrosis, and longer survival after surgery	[226]
Head and Neck Cancer (HNC)	*Pro-tumorigenic* Elevated hGIIA in HNC serum (small numbers). Levels correlate with OS on combined analysis of all 10 malignancies in the study. Elevated sPLA_2_ activity in oral squamous cell carcinoma (OSCC) tissue. hGIIA knockdown in OSCC-derived xenograft reduces tumor volume associated with decreased JNK/c-JUN signaling. Single-cell spatial transcriptomic analysis identifies hGIIA as a marker for a subset of OSCC CAFS that potentially activates pro-tumorigenic α6β4 signaling through IGF-1 signaling in an aggressive population of epithelial cells.	[165,227,228,229]
	*Anti-tumorigenic* Single-cell spatial transcriptomic analysis suggests the hGIIA positive population of OSCC CAFS also activate IGF-1 receptor signaling leading to inactivation of FOXO1 causing uncontrolled oxidative stress in the aggressive epithelial cell population.	No Significant Reports
Lung Cancer	*Pro-tumorigenic* hGIIA is a potential lung cancer biomarker. Downregulation of hGIIA decreases cell proliferation, and xenograft tumor growth associated with NF-κB downregulation. Tumor hGIIA expression suggests K-Ras mutant and squamous cell tumors are potential targets. hGIIA maintains a malignant, therapy-resistant subpopulation of cancer stem cells. hGIIA inhibition reduces tumorsphere formation. hGIIA is a ligand for the EGFR receptor family resulting in increased EGFR/Her2 signaling.	[230,231,232,233,234,235]
	*Anti-tumorigenic* No significant reports	No Significant Reports
Ovarian Cancer (OC)	*Pro-tumorigenic* sPLA_2_ activity, hGIIA mRNA and protein detected in OC effusions and macrophage. Expression correlates with LPA concentrations and is reduced post-chemotherapy. Arachidonic acid detected at high levels (up to 35 mM) and associated with short relapse-free survival.	[236,237,238,239]
	*Anti-tumorigenic* No significant reports	No Significant Reports

## Data Availability

No new data were created or analyzed in this study. Data sharing is not applicable to this article.

## Abbreviations

The following abbreviations are used in this manuscript:

AR	Androgen receptor
ARG1	Arginase-1
BM-PCa	Bone-metastatic prostate cancer
CAFs	Cancer-associated fibroblasts
CCL3	C-C motif chemokine ligand 3
CCL5	C-C motif chemokine ligand 5
CHD1	Chromodomain helicase DNA binding protein 1
COX-2	Cyclooxygenase-2
CRPC	Castration-resistant prostate cancer
CTLs	Cytotoxic T lymphocytes
CXCR4	C-X-C motif chemokine receptor 4
DCs	Dendritic cells
EMT	Epithelial–mesenchymal transition
ERK	Extracellular signal-regulated kinase
EVs	Extracellular vesicles
Gal-1	Galectin-1
hGIIA	Human group IIA secreted phospholipase A2
HIFs	Hypoxia-inducible factors
IKK	IκB kinase
iNOS	Inducible nitric oxide synthase
IL-1β	Interleukin-1 beta
IL-2	Interleukin-2
IL-6	Interleukin-6
LCK	Lymphocyte-specific protein tyrosine kinase
LPA	Lysophosphatidic acid
LPS	Lipopolysaccharide
mCRPC	Metastatic castration-resistant prostate cancer
MCs	Mast cells
MDSCs	Myeloid-derived suppressor cells
meCAFs	Metabolic cancer-associated fibroblasts
MHC	Major histocompatibility complex
MICA	Major histocompatibility complex class I chain-related protein A
MICB	Major histocompatibility complex class I chain-related protein B
MMP-9	Matrix metalloproteinase-9
Mo-DCs	Monocyte-derived dendritic cells
Mo-MDSCs	Monocytic myeloid-derived suppressor cells
NETs	Neutrophil extracellular traps
NF-κB	Nuclear factor kappa B
NK	Natural killer
NKA	Natural killer cell activity
NKG2D	Natural killer group 2 member D
NOX2	NADPH oxidase 2
OS	Overall survival
PA	Phosphatidic acid
PCa	Prostate cancer
PCSC	Prostate cancer stem cell
PD-1	Programmed cell death protein 1
PD-L1	Programmed death-ligand 1
PDAC	Pancreatic ductal adenocarcinoma
PGE_2_	Prostaglandin E_2_
PGD_2_	Prostaglandin D_2_
PGJ_2_	Prostaglandin J_2_
PI3K	Phosphoinositide 3-kinase
PLA_2_	phospholipase A_2_
*PLA2G2A*	Phospholipase A_2_ group IIA gene identifier
PLZF	Promyelocytic leukemia zinc finger protein
PMN	Polymorphonuclear
PMN-MDSCs	Polymorphonuclear myeloid-derived suppressor cells
PSA	Prostate-specific antigen
RNS	Reactive nitrogen species
SDF-1	Stromal cell-derived factor-1
sPLA_2_	Secreted phospholipase A_2_
sPLA_2_-IIA	Secreted phospholipase A_2_ group IIA
sPLA_2_s	Secreted phospholipases A_2_
STAT3	Signal transducer and activator of transcription 3
TAMs	Tumor-associated macrophages
TANs	Tumor-associated neutrophils
TECs	Tumor endothelial cells
TGF-β	Transforming growth factor beta
Th2	T helper 2
TIM-3	T-cell immunoglobulin and mucin domain-containing protein-3
TLSs	Tertiary lymphoid structures
TME	Tumor microenvironment
TNF	Tumor necrosis factor
T*_reg_*	Regulatory T cells
ULBPs	UL16-binding proteins
VEGF	Vascular endothelial growth factor
YAP	Yes-associated protein
